# Formation of actin mesh structures and alpha-smooth muscle actin dynamics in fibroblasts contribute to dermal regeneration in mouse fetus

**DOI:** 10.1371/journal.pone.0331006

**Published:** 2025-09-08

**Authors:** Kento Takaya, Qi Wang, Yuka Imbe, Hiroyuki Nobusue, Keisuke Okabe, Shigeki Sakai, Noriko Aramaki-Hattori, Kenjiro Hanaoka, Hideyuki Saya, Kazuo Kishi

**Affiliations:** 1 Department of Plastic and Reconstructive Surgery, Keio University School of Medicine, Tokyo, Japan; 2 Faculty of Pharmacy, Keio University, Minatoku, Tokyo, Japan; 3 Fujita Cancer Center, Fujita Health University, Aichi, Honshu, Japan; University of Vermont College of Medicine, UNITED STATES OF AMERICA

## Abstract

In adult mammals and other highly developed animals, incomplete wound healing, scar formation, and fibrosis occur. No treatment for complete tissue regeneration is currently available. However, in mice, at up to 13 days of gestation, early embryonic wounds regenerate without visible scarring. In mouse fetuses, actin cable formation at the epidermal wound margin contributes to regeneration after wounding; however, the relationship between actin behavior and dermal regeneration or scar formation by myofibroblasts is unknown. In the present study, we observed actin dynamics in the wound dermis of mouse fetuses and investigated fibroblast and alpha-smooth muscle actin (α-SMA) properties involved in the switch between regeneration and scar formation in the dermis. In the wound healing process of mouse fetuses, actomyosin bundles develop and contract in a mesh-like pattern in different parts depending on the developmental stage, i.e., in the dermis of E13 (regeneration) and in the fascia of E15 and later (scar formation). Furthermore, in E13 dermal fibroblasts, α-SMA is present in the cytoplasm independently of actin, but in E15 and later myofibroblasts, TGFβ-1 stimulation causes the distribution of α-SMA and actin to coincide, and in E17, when dermal scarring occurs, α-SMA is expressed particularly in the nucleus. The results indicate that reticular contraction by actomyosin is involved in dermal regeneration, and that the discrepancy in the localization of actin and α-SMA in fibroblasts is necessary. The findings may contribute to effective wound regeneration therapy.

## Introduction

Scar formation is a natural response of mature mammalian skin to damage. Currently, no therapies exist for complete tissue regeneration. In contrast, during the early stages of pregnancy, the injured fetal skin can regenerate without scar formation [1,2]. Analyzing fetal wound healing patterns may provide insights for therapeutic replication of the fetal healing phenotype of damaged human skin; however, the exact mechanism of scarless wound repair in human skin is currently unknown.

The profile of proteoglycans, collagen, and growth factors, as well as the degree of inflammation in fetal wound differ from those in adult wound. The under-differentiated state of fetal skin is probably an important feature involved in repair without scarring and has long been the focus of scientific attention [3–5]. In particular, it has been shown that during the development of mouse fetus, wounds developing between embryonic day 15 (E15) and E16 heal without histological fibrosis. Furthermore, our earlier studies have established that the skin regenerates without leaving a visible scar in the early phases of mouse development, specifically up to E13, along with restoration of the skin surface texture, dermal structure, and all skin appendages [6–8]. These observations suggest that distinct healing mechanisms in E13 and later-stage embryonic skin are necessary for complete regeneration without scar formation. However, the underlying molecular details are elusive.

Martin, et al. [9] proposed that healing in a mammalian fetus involves the formation of fibrous actin cables at wound margins that function as a contractile “drawstring” for epidermal regeneration. By contrast, it has been proposed that keratinocytes migrate and crawl over the exposed connective tissue via filopodia to facilitate healing of the epidermis in adult animals. In mouse fetuses, until E13, when the epidermis is regenerated, actin filament bundles are formed at the wound edge, and the actin filaments are maintained in a state where they are connected between cells by E-cadherin [7,8]. Conversely, in the period when regeneration does not occur, epidermal cells migrate to the wound site with filopodia composed of actin. However, the involvement of actin filament bundles in the dermis and fascia during the transition process from skin regeneration to scar formation is not clear, and we hypothesized that the changes in actin dynamics may also occur in the dermis.

Another theory of dermal regeneration focuses on the heterogeneity of dermal fibroblasts. Myofibroblasts, absent in normal tissue but active during wound healing, are critical for collagen secretion, microfilament formation, and scarring. Fibrotic disease states are characterized by the progressive migration of abnormally large numbers of active myofibroblasts [10–12]. Mature myofibroblasts have smooth muscle cell-like contractile properties, differentiate from fibroblasts upon stimulation with transforming growth factor-beta 1 (TGF-β1), and express alpha-smooth muscle actin (α-SMA) [13,14]. In mouse subcutaneous fibroblasts, the intracellular distribution of α-SMA and its interaction with the nucleus have been suggested to be critical for mechanical signaling from the cell periphery to the nucleus [15]. This has also been linked to the fibrosis-inducing environment, such as short-term mechanical stretching with reversible cytoskeletal remodeling, characterized by extensive cell spreading and foliar pseudopodia formation [16]. However, the behavior of myofibroblasts in relation to skin regeneration during the developmental stage remains unclear.

Accordingly, this study aimed to characterize the role of fibroblasts in the regeneration of fetal dermal structures by observing α-SMA distribution in mouse embryonic fibroblasts. We investigated the involvement of fibroblast from different layers of embryonic skin in actin cable formation and α-SMA distribution during the complete regeneration of dermal structures using an original mouse embryonic wound healing model reported previously [7,8]. The findings of this study could contribute to the development of therapies for complete wound regeneration in humans.

## Materials and methods

### Animals

#### Ethical considerations.

The research protocol was reviewed and approved by the Institutional Animal Care and Use Committee of the Keio University School of Medicine (approval number: 20170914). All experiments were conducted in accordance with the institutional guidelines for animal experiments at the Keio University School of Medicine.

#### Fetal wound healing model.

The mouse fetal wound healing model was established as previously reported [7,8]. Briefly, 8-week-old female ICR mice (Sankyo Lab Service, Tokyo, Japan) were used in this study. Maternal mice weighed an average of 24 g. Five mice were used for fetal surgery at each time point, i.e., a total of 10 maternal mice were used. Two mother mice were reared per cage. Mice were housed in ventilated cages in temperature-controlled rooms (21 ± 1 °C) with a 12 h light/12 h dark cycle. Food and water were freely available. The fetus was designated as E0 when a vaginal plug was visible; at E13 and E15, the fetus was wounded using an operating microscope. At each time point, a minimum of four fetuses were operated on per mother. Pregnant mice were sedated with 3% v/v isoflurane inhalation anesthesia, their abdominal walls were incised to expose the uterus, and the myometrium, amnion, and yolk sac were incised. Then, using surgical micro-scissors, a full-layer incision of approximately 2 mm in length was made in the lateral thoracic region of the fetus. At E13, the myometrium was left open and unsutured to prevent preterm delivery or uterine rupture due to increased intrauterine pressure; at E15, the myometrium was sutured with 9−0 nylon after wounding, the uterus was returned into the abdominal cavity, and the abdomen was closed. In addition, 1 mg/g body weight (dissolved in 100 µL of saline) of ritodrine hydrochloride (FUJIFILM Wako Pure Chemical, Osaka, Japan) was administered intraperitoneally to prevent uterine rupture immediately before the abdominal wound was closed. The peritoneum and skin were then sutured using 5−0 nylon thread. Maternal mice were euthanized by cervical dislocation, and fetuses were harvested 24 h after wounding. Fetal skin was harvested and fixed in 4% paraformaldehyde (PFA) for 24 h. For immunostaining, the fixed tissues were immersed in 20% sucrose in phosphate-buffered saline (PBS), frozen, embedded in OCT compound (Sakura Finetek Japan Co., Ltd., Tokyo, Japan), and sliced into 7-mm sections. We observed at least three fetuses obtained from different mothers, which were immunostained under the same conditions.

#### Fibroblasts from the fetal dermis and fascia.

The unwounded fetal skin at E13, E14, E15, E16, and E17 was cut in layers and separated into the dermis and fascia under a microscope. Each sample was cut into small pieces with a scalpel, and the pieces were incubated in 10 mL low-glucose Dulbecco’s Modified Eagle’s medium (FUJIFILM Wako Pure Chemical) supplemented with 10% fetal bovine serum (Thermo Fisher Scientific, Waltham, MA, USA) and 1% penicillin/streptomycin (Thermo Fisher Scientific) at 37 °C and 5% CO_2_ overnight. Then, 7 mL of the medium was added, and the medium was changed every three days to obtain fibroblasts. As the skin was too thin to separate into the dermis and fascia at E13, the tissue below the dermis was cut and cultured, and the obtained cells were used as dermal fibroblasts at E13.

### Cell TGF-β stimulation and scratch assays

Separated dermis and fascia were explanted into separate plastic dishes for primary culture of local mesenchymal cells. After passages 5–7, migration assays were performed on cells. Before each assay, the cells were treated with mitomycin C (NAKARAI TESQUE, INC., Kyoto, Japan) to exclude proliferative effects (10 mg/mL for 3 h at 37 °C).

For the TGF-β1 assay, the cells were cultured on collagen type I-coated dishes until confluence; recombinant mouse TGF-β1 (10 ng/mL; R&D Systems, Minneapolis, MS, USA) was then added, and the cells were cultured for three days at 37 °C and 5% CO_2_ with 95% relative humidity.

For the scratch assay, the cells were grown to confluence on plastic dishes, and a 500-mm scratch was then made with a pipette tip. The cells were cultured in Dulbecco’s modified Eagle’s medium (FUJIFILM Wako Pure Chemical, Osaka, Japan) for up to 24 h at 37 °C and 5% CO_2_ with 95% relative humidity. After each assay, the cells were analyzed by immunostaining.

### Immunostaining

The cells were fixed in 4% PFA for 10 min at room temperature (20–25 °C) and then washed once for 30 s in PBS. To stain the cells in the non-adherent state, they were seeded on 1% agarose-coated dishes, fixed in 4% PFA for 20 min, centrifuged, and the resulting pellet was dropped onto glass slides, smeared, and allowed to air-dry. Tissue sections were thinned to 7 µm and air-dried for 1 h. The samples were incubated with 3% bovine serum albumin/PBS as the blocking solution for 1 h at room temperature (20–25 °C). For immunostaining, the primary antibody was diluted in the blocking solution, and the cells and tissues were incubated overnight at 4 °C in the blocking solution with the antibody. The following primary antibodies used were rabbit anti-N-cadherin antibody (ab76011, 1: 200; Abcam, Cambridge, UK), rabbit anti-integrin-b1 antibody (ZRB1230, 1:100; Sigma-Aldrich, St. Louis, MO, USA), rabbit anti-myosin antibody (HPA040902, 1:200; Sigma-Aldrich), and rabbit anti-alpha-SMA antibody (ab5694, 1:100; Abcam). The samples were washed thrice with PBS and then incubated in a solution of the secondary antibody anti-rabbit Alexa Fluor 555 (Thermo Fisher Scientific, 1:200) diluted with PBS for 1 h at room temperature (20–25 °C). After washing thrice with PBS, the actin, nuclei, and plasma membranes were stained by incubating with Acti-stain 488 phalloidin (PHDG1-A, 1:200; Cytoskeleton, Inc., Denver, CO, USA), DAPI (1:500; Thermo Fisher Scientific), and CellMask Deep Red plasma membrane stain (1:1000; Thermo Fisher Scientific), respectively, for 1 h at room temperature (20–25 °C). The slides were sealed on glass slides using ProLong Gold (Thermo Fisher Scientific). All slides were viewed under a confocal laser scanning microscope (FLUO-VIEW FV3000; Olympus, Co., Ltd., Osaka, Japan) with Z-stack. The filament analysis of actin was performed using the 3D image analysis software Imaris (Zeiss, Oberkochen, Germany), which automatically detects and creates a 3D model based on the width and brightness values of the set filaments to analyze the branching state [18].

### RNA extraction and reverse transcription

Total RNA was extracted from the cells using RNeasy Mini Kit (Qiagen, Hilden, Germany), according to the manufacturer’s instructions. Total RNA was mixed with PrimeScript RT Master Mix (Takara Bio, Shiga, Japan). The annealing step was performed in a T100TM thermal cycler (Bio-Rad Laboratories, Inc., Hercules, CA, USA) at 25 °C for 5 min, DNA was synthesized at 55 °C for 10 min, and reverse transcriptase was heat-inactivated at 80 °C for 10 min to obtain cDNA.

### Gene expression analysis

Real-time quantitative polymerase chain reaction was performed using the Applied Biosystems 7500 Fast Real-Time PCR System (Thermo Fisher Scientific). The fluorescence of each sample was measured throughout the course of 40 cycles, at the end of each cycle. Gene expression was analyzed using α-SMA probe (Mm01546133_m1; Thermo Fisher Scientific) and PCR master mix (4352042; Thermo Fisher Scientific), according to the manufacturer’s instructions. *ACTB* assay (Mm 02619580_g1; Thermo Fisher Scientific) was used as an endogenous control for data normalization. Gene expression levels of proliferating cells were used as the baseline, and relative gene expression levels were determined using the ΔΔCt method.

### Western blotting

Total protein was extracted from cells using RIPA Lysis Buffer (Santa Cruz Biotechnology, Santa Cruz, CA, USA), according to the manufacturer’s instructions. Extraction of cytoplasmic and nuclear protein fractions was performed using LysoPure Nuclear and Cytoplasmic Extractor Kit (FUJIFILM Wako Pure Chemical), according to the manufacturer’s protocol. Each sample (40 mg) was then resolved by electrophoresis on 10% polyacrylamide Mini-PROTEAN TGX Precast Gels (Bio-Rad Laboratories, Inc.) at 200 V and 50 mA. The cells were then transferred to polyvinylidene difluoride membranes (Millipore, Bedford, MA, USA) using Trans-Blot Turbo Transfer System (Bio-Rad Laboratories, Inc.). The membranes were blocked with 3% nonfat milk for 1 h at room temperature (20–25 °C), and the antigen–antibody reaction was performed using SNAP i.d. 2.0 system (Merck, Darmstadt, Germany), following manufacturer’s instructions. Rabbit anti-alpha-SMA antibody (ab5694, 1:100; Abcam) and rabbit anti-b-actin antibody (13E5, 1:1000; Cell Signalling Technology, Danvers, MS, USA) were used as primary antibodies, and goat anti-rabbit IgG H&L (horseradish peroxidase) (ab205718, 1:5000; Abcam) as secondary antibody. After final wash of the membranes, immunoreactive protein bands were visualized using an electrochemiluminescence detection kit (Pierce Biotechnology, Rockford, IL, USA), according to the manufacturer’s protocol. The expression level of beta-actin was used as a loading control. The bands were imaged using a chemiluminescence imager (ImageQuant LAS4000 mini; GE Healthcare, Chicago, IL, USA). Image analysis was performed using ImageJ software (Ver. 1.53; National Institutes of Health, Maryland, USA). Each experiment was repeated thrice.

### Quantification and statistical analysis

Statistical analysis was performed using GraphPad Prism (version 5.0; San Diego, CA, USA) or SPSS 22.0 (Chicago, IL, USA). The one-way analysis of variance (ANOVA) and Tukey’s post-hoc test were used to compare differences. In other words, we compared each of the two groups, with and without stimulation, in each cell. The statistical significance was set at *P* < 0.05. Each experiment was performed at least three times; all statistical details of the experiments can be found in figure legends.

## Results

### The dermis of E13 and fascia of E15 contain a meshwork of actin at the wound margin

First, actin staining and filament analysis were performed to examine actin expression in the dermis and fascia during wound healing in E13 and E15 embryonic mice. These time points were selected based on switching of the pattern of wound healing from complete wound regeneration, including skin texture by actin cables (early embryonic type), to adult-type cell migration that leaves a visible mark (late embryonic type) [7,8].

**Fig 1:**
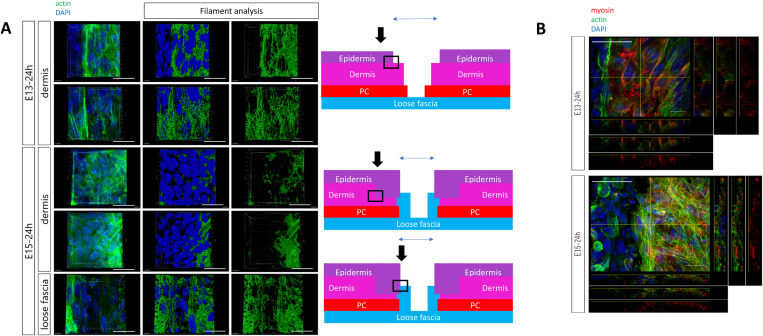
The dermis of E13 and fascia of E15 contain a meshwork of actin at the wound margin. (A) Analysis of actin fibers in the dermis and fascia of E13 and E15 wound margins. Green and blue fluorescence signals correspond to phalloidin (actin) and diaminino-2-phenylindole (DAPI; nucleus), respectively. For filament analysis, 3D filament models (middle column) were constructed from immunostaining images (left column) and only actin filaments were extracted (right column). The upper row of this two-column chart represents the superficial layer of the dermis, and the bottom row represents the deep layer of the dermis. Scale bar = 50 mm. The images are representative of three technical replicates and three biological replicates. (B) Myosin expression in E13 and E15 wound margins. Red, green, and blue fluorescence signals correspond to myosin, actin, and DAPI (nucleus), respectively. Scale bar = 50 mm. In the schematic on the right, the observed areas of the wound are indicated by arrows and black squares. PC; panniculus carnosus muscle.

We have previously shown that actin exhibits a single cable-like structure along the cell membrane in the epidermis at the wound margin of the E13 mice and contributes to complete regeneration of epidermal texture [7,8]. However, in the dermis of the E13 wound margin observed in the current study, actin was present in bundles rather than cables, forming a network at the wound margin (Fig 1A). In contrast, in the dermis of the E15 wound, the actin network structure was not observed and actin was expressed non-specifically in a plane-like manner, whereas in the fascia (deeper layer), the mesh-like structure of actin was present, as seen in the E13 dermis. When the localization of myosin, which binds actin filaments and is involved in cytoskeletal formation, was observed, myosin molecules were confirmed to be present on the mesh-like actin filaments in the E13 dermis and E15 fascia layers at the wound edge. (Fig 1B). The fact that actin forms a mesh-like network of actomyosin in the dermis of E13, where wounds regenerate completely, but not in the dermis of E15, where wounds do not regenerate, but only in the fascia, suggests that this may play a role in the difference in wound healing patterns.

### Fibroblasts in fetal mouse skin form actomyosin

To investigate the cellular mechanisms contributing to actin dynamics observed in the dermis and fascia of fetal mouse wounds, we investigated the relationship between actin and myosin behavior and cell adhesion molecules in E13, E15, and E17 dermal and fascial fibroblasts. Specifically, we observed the dynamics of fibroblasts in a wound-mimicking state by inducing fibroblast differentiation into myofibroblasts and activating wound healing signals by administering TGF-β1.

**Fig 2:**
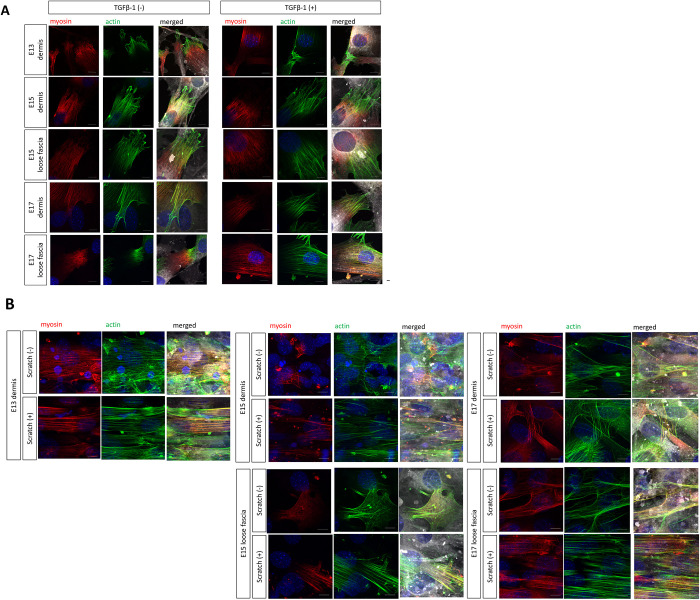
Fibroblasts in fetal mouse skin form actomyosin, as determined by the analysis of the expression of actomyosin in dermal and myofascial fibroblasts from mouse fetuses. (A) TGF-β1 stimulation alters the distribution of actin and myosin. Scale bar = 10 mm. Red, green, white, and blue fluorescence signals correspond to myosin, phalloidin (actin), plasma membrane, and DAPI (nucleus), respectively. (B) Changes in the distribution of actin and myosin, as determined by the scratch assay that mimicked wound stimulation. This shows the state of the scratch after 24 h. Scale bar = 50 mm. Red, green, white, and blue fluorescence signals correspond to myosin, actin, plasma membrane, and DAPI (nucleus), respectively. The lower image is an enlargement of white square area in the upper column.

E13: In the absence of TGF-β1 stimulation, the location of myosin was unrelated to that of actin filaments in dermal fibroblasts, but in the presence of TGF-β1 stimulation, myosin was aligned slightly with actin filaments. E15: In the absence of TGF-β1, myosin was present independently of actin filaments in both the dermis and fascia fibroblasts. However, in the presence of TGF-β1, both the dermis and the fascia fibroblasts showed co-localization of actin and myosin. For E17, while fascial fibroblasts showed co-localization of actin and myosin in response to TGF-β1 stimulation, as in E15, the dermal fibroblasts showed co-localization of actin and myosin in some cells, even in the absence of TGF-β1 stimulation, and the co-localization tended to increase with an increase in stimulation (Fig 2A). Similar results were obtained in another model that mimics wounds, the scratch pressure model. Specifically, in E13, myosin, which is scattered throughout the cytoplasm, clearly co-localized with actin in response to scratch stimulation. As with TGF-β1 stimulation, in E15, both dermal fibroblasts and fascia fibroblasts showed clear co-localization of actin and myosin due to scratch stimulation. In E17, as with TGF-β1 stimulation, the co-localization of actin and myosin, which is observed even in the steady state in dermal fibroblasts, increased due to scratch stimulation, and in fascia fibroblasts, co-localization, which is not clear in the steady state, appeared due to scratch stimulation (Fig 2B). The co-localization of these actin filaments and myosin indicates the formation of actomyosin fibers, which are a characteristic of myofibroblasts. This suggests that fibroblasts from either fetus can differentiate into myofibroblasts upon wound stimulation.

### α-SMA expression dynamics in dermal and fascial fibroblasts accompany wound healing patterns in fetal mice

The experimental results thus far indicate that embryonic fibroblasts at each developmental stage form actomyosin in response to wound stimuli but the explanation for the functional differences is not satisfactory. Previous studies have shown that the location of α-SMA, a marker of mature myofibroblasts, and stress fibers modulate their function in some fibroblasts [17]. We examined the localization of actin and α-SMA in E13 dermis, E15 dermis and fascia, and E17 dermis and fascia fibroblasts to detect their possible involvement in wound regeneration and healing.

**Fig 3:**
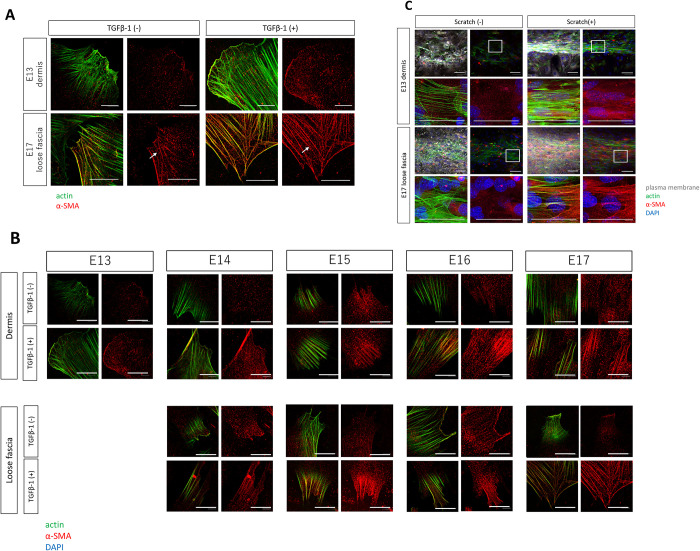
Relationship between α-SMA actin fiber localization in fetal mice cells and wound stimulation. (A) Altered α-SMA distribution in dermal and fascial fibroblasts at various developmental stages in response to TGF-β1 stimulation. Scale bar = 20 mm. Red and green fluorescence signals correspond to α-SMA and actin, respectively. (C) α-SMA distribution and actin expression in E13 dermal and E17 fascial fibroblasts in the scratch assay. Scale bar = 10 mm. Red, green, white, and blue fluorescence signals correspond to α-SMA, phalloidin (actin), plasma membrane, and DAPI (nucleus), respectively.

First, we compared the localization of α-SMA in the two cells with the most opposite properties in wound healing during embryonic development: fibroblasts of E13 dermis, where wounds regenerate, and fibroblasts of E17 fascia, which have been shown to be involved in scar formation and correspond to adult animal fibroblasts in a previous study [7,8]. In fibroblasts of E13 dermis, α-SMA was barely present along actin fibers with or without TGF-β1 stimulation, whereas in fibroblasts of E17 fascia, it was localized along actin fibers upon TGF-β1 stimulation (Fig 3A). We then examined α-SMA localization in all dermal and fascial fibroblasts at all developmental stages in the presence or absence of TGF-β1 stimulation. In the steady state, in which the cells have not been treated with TGF-β1, the presence of α-SMA along actin filaments was not detected at most time points, and only slight co-localization was observed in the E17 myofibroblasts. In E13 dermal fibroblasts and E14 dermal and fascia fibroblasts, there was no co-localization of actin and α-SMA even after TGF-β1 treatment. In E15 and E16, there was only a small amount of co-localization of α-SMA and actin in dermal fibroblasts after TGF-β1 treatment. More co-localization was observed in E17. In the fascia fibroblasts from E15 onwards, a-SMA and actin were co-localized by TGF-β1 treatment (Fig 3B).

However, in fibroblasts of E17 fascia, localized α-SMA expression along actin fibers was observed under scratch stimulation, similar to TGF-β1 stimulation. (Fig 3C). We observed that α-SMA was absent along actin in E13 dermis at the wound edge 24 h after wound healing, although it was present in E15 fascia, in an *in vivo* fetal mouse wound model (S1 Fig).

**Fig 4:**
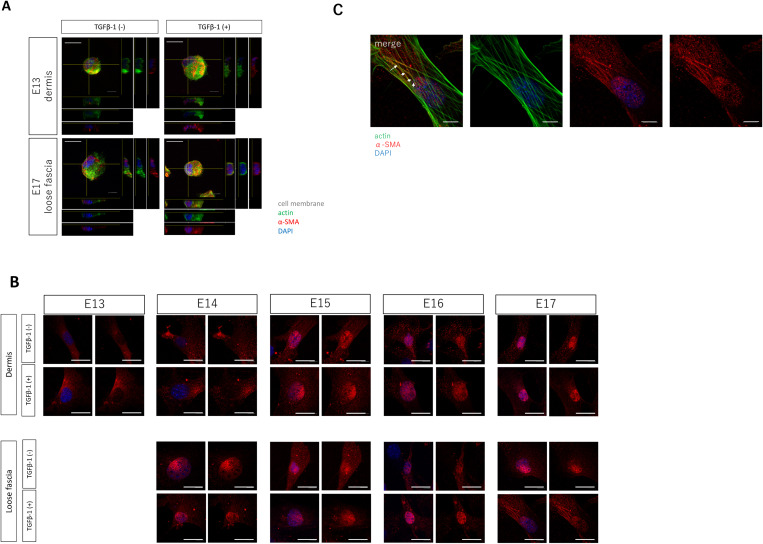
Relationship between nuclear translocation of α-SMA and differentiation into myofibroblasts in fetal mice fibroblasts. (A) Localization of α-SMA and actin in fibroblasts in the non-adherent state. Scale bar = 20 mm. Red, green, and blue fluorescence signals correspond to α-SMA, actin, and DAPI (nucleus), respectively. (B) Altered α-SMA distribution in dermal and fascial fibroblasts at various developmental stages in response to TGF-β1 stimulation. Scale bar = 20 mm. Red and green fluorescence signals correspond to α-SMA and actin, respectively. (C) Distribution of actin and α-SMA in E17 fascial fibroblasts after TGF-β1 stimulation. Scale bar = 20 mm. Red, green, and blue fluorescence signals correspond to α-SMA, actin, and DAPI (nucleus), respectively. Arrows indicate the localization of α-SMA in line with the actin fibers.

While the association between fibrosis (myofibroblast differentiation) and actin fiber localization of α-SMA was evident, we investigated whether TGF-β1 stimulation affects the α-SMA localization and whether α-SMA behavior itself influences fibroblast function by placing fibroblasts in a non-adherent state, to achieve stress fiber and cell adhesion unrelated conditions (Fig 4A). In other words, when fibroblasts are adhered, actin is mobilized to form stress fibers. However, when they are not adhered, actin is not mobilized to form stress fibers. Therefore, by investigating the positional relationship between actin and α-SMA in this state, we investigated the effect of localization itself on cell function. Similar observations in non-adherent cells demonstrated altered nuclear localization of α-SMA protein. In the absence of TGF-β1, fibroblasts of E13 dermis had little or no nuclear expression of α-SMA, while fibroblasts in E17 fascia had α-SMA in their nuclei in normal state. When TGF-β1 was added, α-SMA was found to be expressed in a portion of the nucleus in E13, and even more α-SMA was found in nuclei of the cells in E17 than in the state without TGF-β1 stimulation. It was speculated that α-SMA migrated into the nucleus upon TGF-β1 stimulation and involved in scar formation. In addition, α-SMA migrated to the nucleus without TGF-β1 stimulation in E17 fascial fibroblasts, suggesting that these cells are susceptible to scar formation (Fig 4C).

Observation of fibroblasts from E13 to E17 at all developmental stages revealed that, first, in dermal fibroblasts, there was little sign of migration of α-SMA into the nucleus in E13 and E14, with only minimal accumulation observed when present (Fig 4B). In contrast, in dermal fibroblast after E15, α-SMA was found to be expressed in the nucleus, and this accumulation remained unchanged or was partially enhanced by TGF-β1 stimulation. In fascial fibroblasts from E14 to E17, α-SMA was expressed in the nucleus at all developmental stages, and this accumulation was also unchanged or partially enhanced by TGF-β1 stimulation.

**Fig 5:**
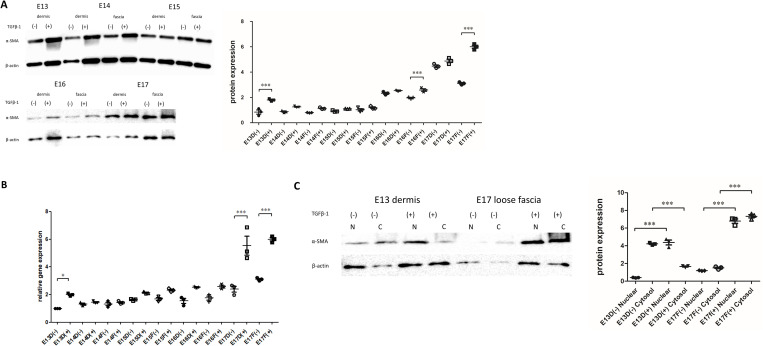
Quantitative evaluation of α-SMA expression in fetal mice fibroblasts. (A) Western blot analysis of α-SMA expression in mice fibroblasts at different developmental stages and under different stimulation conditions. *P < 0.05. All experiments were independently repeated three times; the values are presented as the mean ± standard deviation (SD). One-way analysis of variance (ANOVA) and Tukey’s post-hoc test were used to compare differences between the groups. (B) Analysis of relative mRNA levels of the α-SMA gene in developing mouse fibroblasts by real-time quantitative polymerase chain reaction. (C) Western blot analysis of α-SMA expression in the nucleus and cytoplasm of E13 dermal and E17 fascial fibroblasts. *N* nucleus, *C* cytoplasm. *P < 0.05. All experiments were independently repeated three times; the values are presented as the mean ± standard deviation (SD). One-way analysis of variance (ANOVA) and Tukey’s post-hoc test were used to compare differences between the groups.

Quantification of α-SMA expression in fibroblasts at each developmental stage revealed that TGF-β1 stimulation at the protein level increased α-SMA expression in all cells. Upregulation of α-SMA by TGF-β1 was also significant in E13 dermis and E17 fascia (Fig 5A). Increased mRNA levels of α-SMA in response to TGF-β1 stimulation were found to be significant in the cells of E13 dermis, E16 dermis and fascia, and E17 dermis and fascia (Fig 5B).

Analysis of proteins in the nuclear and cytoplasmic fractions indicated that α-SMA was predominantly expressed in the cytoplasm of E13 dermal fibroblasts in the absence of TGF-β1, but its levels were significantly increased in the nucleus when TGF-β1 was added (Fig 5C). However, in the absence of TGF-β1 stimulation, α-SMA expression in E17 fascia fibroblasts was higher in the nucleus than in the cytoplasm, with no significant change in the distribution or increase or decrease in the expression observed upon TGF-β1 stimulation.

In summary, in the mouse embryonic development stage, a mesh structure formed by actin was formed in the dermis during the regeneration period and in the fascia thereafter. This mesh structure was potentially a stress fiber formed by actomyosin. Furthermore, when the fibroblasts at each stage were observed, α-SMA, a marker for myofibroblasts, was present in the cytoplasm of fibroblasts in the E13 dermis during wound regeneration, which contributes to the formation of the mesh structure, and its expression did not coincide with that of actin. In dermal fibroblasts from E15 onward, TGF-β1 stimulation affects α-SMA and actin distribution. In myofibroblasts, α-SMA is expressed in the nucleus even in the steady state, and especially in E17, when scar formation occurs. The findings suggest that the nuclear translocation of α-SMA and changes in actin behavior in mice during the scar formation period may regulate the properties of fibroblasts that form scars.

## Discussion

Scar tissue is characterized by (1) dermal fibrosis, (2) changes in skin texture, (3) loss of skin appendages, and (4) alterations in skin pigmentation [18,19]. Therefore, observing the presence or absence of regeneration based on the skin texture and dermal structure except the color tone is crucial for assessing scars from a morphological perspective. Various studies have been conducted to achieve complete skin regeneration, including all of the above factors. Although the function of actin in epidermal regeneration has been previously established through our unique wound healing model using fetal mice [7,8], the mechanism of dermal structure regeneration and involvement of fibroblasts in this process remain unresolved.

The results of the present study suggest that a mesh structure formed by actomyosin bundles is formed in the dermis in E13 when complete wound regeneration occurs without leaving a visible wound mark and histologic scar tissue. Stress fibers formed by actomyosin bundles normally take on a cable-like structure, and a cable (actin cable) is formed specifically in the epidermis at E13; however, in the dermis, the structure is mesh-like [8]. In other words, the contraction of the epidermis by the actin cable structure, like a drawstring being tightened, rather than the behavior of the dermis, may contribute to the regeneration of complete skin, such as in E13. By contrast, actomyosin bundles form a mesh-like structure in the fascia at the wound margins in E15, where visible wound mark are present but do not histologically generate fibrotic scar tissue like in adults. This pattern of actomyosin formation is consistent with the transition between wound regeneration and repair, and may explain why the epidermis and dermis are positioned differently after E13 and E14, highlighting one of the histological differences between these two stages.

The contractile forces of actin and actomyosin during the wound healing process are important for the mechanical properties of the epithelium because of its multicellular structure. Importantly, the structure of the actin cytoskeleton that mediates these contractile forces varies widely among cell types. In *Drosophila*, actomyosin forms a contractile ring around the cell population on the epithelial sheet during the early stages of tracheal cord development [20]. However, during the early stages of *Drosophila* protoderm formation, actomyosin forms contractile mesh patches covering the apical surface of the cell population, and these structures have been reported to provide different deformation properties [21]. Thus, intracellular contractile structures may be well organized on multiple scales to modulate the active and passive behavior of the tissue in response to different stress types, including mechanical, tensile, compressive, and shear. The regulated positioning of actomyosin mesh structures in wounds during the developmental stages of mouse fetuses observed in this study may be one element of this hypothesis.

Earlier studies have demonstrated that adult wound scars comprise fascia-derived mesenchymal cells [22–24]. Furthermore, in fetal wound healing during the late embryonic period, when the scar remains, fascial mesenchymal cells construct scar tissue as in adult animals, but dermal mesenchymal cells cause dermal regeneration. The differential nature of fibroblasts in the dermis and fascia is speculated to be responsible for regeneration and repair. We explored this in the current study.

During *in vivo* wound healing, we found that α-SMA did not coincide with actin fibers in the dermis of E13 but was present along actin fibers in the fascia of E15. At the E14/E15 transition, dermal fibroblasts exhibited distinct α-SMA dynamics. If the pattern of α-SMA and actin expression coincides with TGF-β1 stimulation after E15, these cells would likely differentiate into myofibroblasts and contribute to scar formation, consistent with the histological fibrosis switch we have previously discovered in the fetal stage [7,8]. Although developing fetal skin contains high levels of all (three) TGF-β isoforms, it retains the inherent ability to heal without scarring [25,26]. Compared to human skin fibroblasts, TGF-β1 stimulation strongly induces α-SMA upregulation in fetal fibroblasts [27]. In the mouse fetus, TGF-β1 stimulation enhances α-SMA expression along actin cables, and nuclear migration of α-SMA occurs in the myometrium at E17 when TGF-β1 stimulates collagen synthesis in fetal fibroblasts [28]. This suggests that fibroblasts that express α-SMA in the cytoplasm, regardless of whether or not they have been stimulated by TGF-β1, i.e., E13 or E14 dermal fibroblasts, may contribute to the healing of early embryos without leaving scars. Conversely, E15-E17 fascia fibroblasts, which show TGF-β1-responsive nuclear translocation of α-SMA expression, may contribute to late-stage scar formation. In a previous study, TGF-β1-responsive E15 cells showed decreased expression of procollagen 1a1, whereas E18 cells showed increased expression of procollagen 1a1 and decreased expression of procollagen 3, suggesting the increased type 3–1 collagen ratio found in fetal wound without scars [29]. The differences in the profiles of these fibroblasts may be related to their site of origin and α-SMA localization.

Interestingly, α-SMA protein and gene expression levels were high in the later stages of development, regardless of whether or not there was a wound stimulus. To date, there have been no reports of the absolute amount of α-SMA expression influencing the function or fate of fibroblasts or myofibroblasts. Our results suggest that both the absolute amount of α-SMA in fibroblasts and changes in its localization (nuclear translocation) may determine clusters of fibroblasts that contribute to scarring.

The lack of clarity on the involvement of α-SMA localization and actin dynamics in collagen synthesis is one limitation of the study. Our findings reveal a link between altered patterns of wound healing during mouse development and actin dynamics in the dermis and the localization of α-SMA in fibroblasts; however, it is unclear how these alter the pattern of collagen production that leads to wound fibrosis. Changes in actin dynamics and wound healing upon the promotion or inhibition of such nuclear localization should be examined in the future. Furthermore, because of species-specific differences in melanocyte layering, hair cycle, immune response, cell signaling, oncogenes, and skin structure between rodents and humans, it is unclear whether the changes in molecular behavior of actin and α-SMA discovered here will contribute to the realization of skin regeneration in humans [30–32]. E13 mouse wounds regenerate up to the layer leading to the panniculus carnosus muscle, corresponding to the human superficial fascia [7,8,33]. Nonetheless, in the present study, only the dermis was observed to contain a contractile structure with actin mesh, indicating that additional investigation is required to regenerate panniculus carnosus muscle. Another possible limitation of this study is that we were unable to control for the sex of fetuses examined in this study, and future studies are needed to determine the sex differences in our findings.

## Conclusions

In conclusion, E13 dermal fibroblasts, which contribute to complete wound regeneration, form an actin mesh that allows dermal contraction and regeneration but does not bind α-SMA. In contrast, actin and α-SMA co-localize in fibroblasts that form scars during the late mouse developmental stage. Therefore, differences in α-SMA activity in the mesh of actin fibers and fibroblasts may affect complete skin regeneration and dermal healing during scar formation.

## Supporting information

S1 Fig Expression and distribution of α-SMA and actin in fetal wound models. Scale bar = 50 mm. Red, green and blue fluorescence signals correspond to α-SMA, actin, and DAPI (nucleus), respectively. In the *in vivo* wound model, α-SMA was absent along actin in E13 dermis at the wound edge 24 h after wound healing, although it was present in E15 fascia.
(TIF)
