# Supplementary figures and images for: Formation of actin mesh structures and alpha-smooth muscle actin dynamics in fibroblasts contribute to dermal regeneration in mouse fetus
Source: PLoS One. 2025 Sep 8;20(9):e0331006. doi: 10.1371/journal.pone.0331006 (PMC12416743; doi:10.1371/journal.pone.0331006)

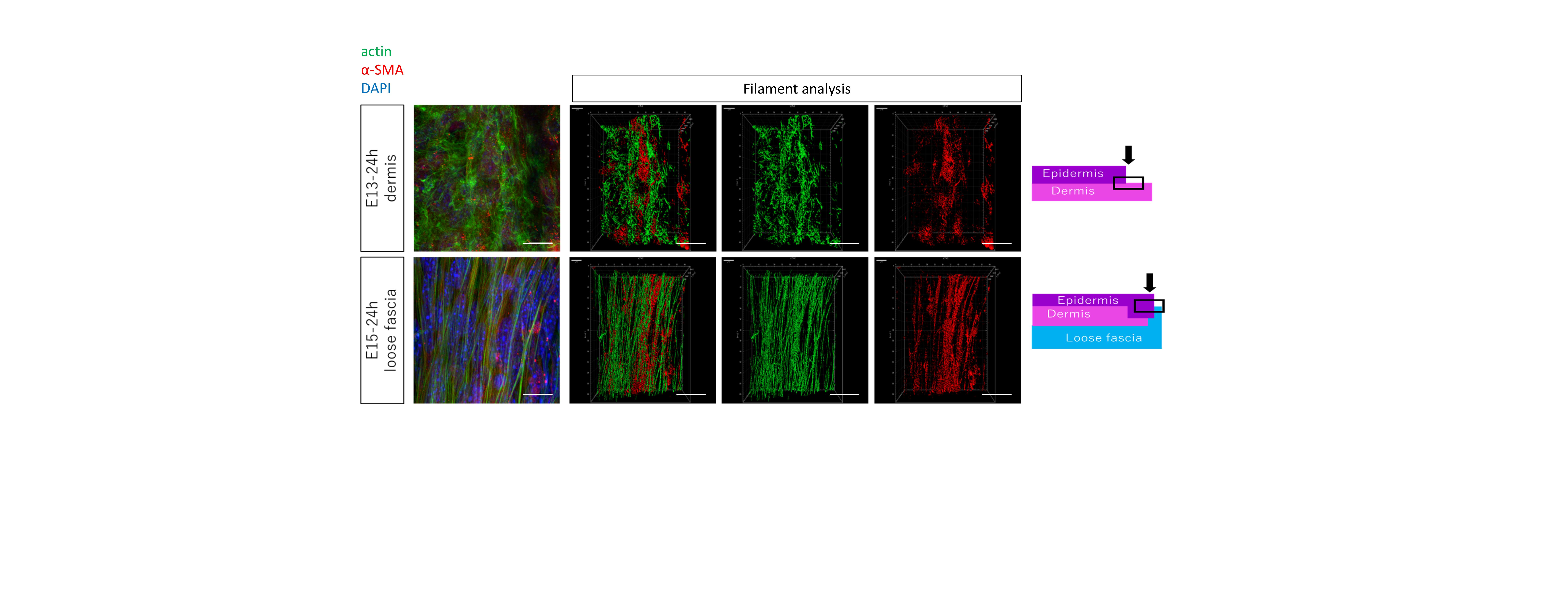

Supplement: S1 Fig — Scale bar = 50 mm. Red, green and blue fluorescence signals correspond to α-SMA, actin, and DAPI (nucleus), respectively. In the in vivo wound model, α-SMA was absent along actin in E13 dermis at the wound edge 24 h after wound healing, although it was present in E15 fascia. (TIF) [file pone.0331006.s001.TIF]
